# Infraglenoidal scapular notching in reverse total shoulder replacement: a prospective series of 60 cases and systematic review of the literature

**DOI:** 10.1186/1471-2474-12-101

**Published:** 2011-05-19

**Authors:** Patrick Sadoghi, Andreas Leithner, Patrick Vavken, Andreas Hölzer, Josef Hochreiter, Georg Weber, Matthias F Pietschmann, Peter E Müller

**Affiliations:** 1 Department of Orthopaedic Surgery, Ludwig-Maximilians-University Munich, Campus Grosshadern, Marchioninistrasse 15, 81377 Munich, Germany; 2 Department of Orthopaedic Surgery, Medical University of Graz, Auenbruggerplatz 5, 8036 Graz, Austria; 3 Department of Orthopedic Surgery, Children's Hospital Boston, Harvard Medical School, Boston MA 02115, USA; 4 Harvard Center for Population and Development Studies, Harvard School of Public Health, Boston, MA 02115, USA; 5 Department of Orthopaedic Surgery, Krankenhaus Barmherzige Schwestern Linz, Seilerstätte 4, 4010 Linz, Österreich

**Keywords:** Inverse shoulder prosthesis, notching, instability, clinical outcome

## Abstract

**Background:**

The impact of infraglenoidal scapular notching in reversed total shoulder arthroplasty (RTSA) is still controversially discussed. Our goal was to evaluate its potential influence on subjective shoulder stability and clinical outcome. We hypothesized that subjective instability and clinical outcome after implantation of RTSA correlates with objective scapular notching.

**Methods:**

Sixty shoulders were assessed preoperatively and at minimum 2-year follow-up for active range of motion and by use of the Oxford instability score, Rowe score for instability, Constant score for pain, Constant shoulder score, DASH score. All shoulders were evaluated on anterior-posterior and axillary lateral radiographic views. These X-ray scans were classified twice by two orthopaedic surgeons with respect to infraglenoidal scapular notching according to the classification of Nerot. Notching was tested for correlation with clinical outcome scores to the evaluated notching.

**Results:**

We found no significant correlation between infraglenoidal scapular notching and clinical outcomes after a mid-term follow-up from 24 to 60 months, but at the final follow-up of 60 months and more, we did see statistically significant, positive correlations between infraglenoidal scapular notching and the Constant pain score as well as active range of motion. At mean follow-up of 42 months (range from 24 to 96 months) we found no significant correlation between subjective instability and infraglenoidal scapular notching.

**Conclusions:**

We conclude that patients' subjective impression on their shoulders' stability is not correlating with radiological signs of infraglenoidal scapular notching. Nevertheless clinical parameters are affected by infraglenoidal scapular notching, at least in the long term

## Background

Infraglenoidal scapular notching in reverse total shoulder arthroplasty is a frequent finding [1-13]. It is related to mechanical impingement by the medial rim of the humeral cup against the scapular neck in adduction and assumed to be an important risk factor for subsequent glenoid loosening [2,3,8,9,14]. The relevance of infraglenoidal scapular notching in terms of a worse clinical outcome, increased polyethylene wear and subsequent local osteolysis, chronic inflammation and subjective satisfaction is still controversially discussed in the current literature [11,15]. Whilst Lévigne et al. [5] do not report a correlation of scapular notching with pain scores and clinical findings, Sirveaux et al. [16,17] showed a negative effect of scapular notching on clinical outcome, at least in terms of the Constant shoulder score. Thus, the influence of infraglenoidal scapular notching on the clinical outcome has still not been fully delineated.

In the present study we evaluated 60 consecutive patients treated with the Delta reverse total shoulder prothesis (DePuy France, Saint Priest CEDEX, France) using clinical and radiological scores with a focus on the influence of infraglenoidal scapular notching and systematically reviewed the related literature.

The aim of the study was to evaluate a potential influence of infraglenoidal scapular notching on stability and clinical outcome at minimum 2-year follow-up in reversed total shoulder arthroplasty.

The first study hypothesis (H_1_) was, that objective infraglenoidal scapular notching correlates with subjective instability analysed by the Oxford instability score and Rowe score for instability. The second study hypothesis (H_2_) was, that objective infraglenoidal scapular notching correlates with clinical outcome, measured by the Constant pain score, Constant shoulder score, DASH score and range of motion (ROM)

## Methods

The study protocol was approved by the responsible, local Institutional Review Board (IRB). All patients included in the present study were operated on by one single surgeon with the Delta reverse ball-and-socket prosthesis (DePuy France, Saint Priest CEDEX, France) between February 2002 and June 2007 without changes in the procedure. The indications for surgery were massive rotator cuff tears with or without massive shoulder arthritis. All patients suffered from so called "pseudoparesis" with no active elevation of the shoulder exceeding 90 degrees, degenerative changes of the glenohumeral joint and/or massive rotator cuff tears. Patients with a minimum of 2 years clinical and radiological follow-up were included in this study. We excluded patients with acute fractures, trauma, or revision arthroplasty from this analysis. These exclusion and inclusion criteria met all patients of this study.

Patients were preoperatively assessed using the Oxford instability score [18], Rowe score for instability [18], Constant score for pain [18], Constant shoulder score [18], and active range of motion. Preoperative radiological evaluation included anterior-posterior and axillary lateral X-ray studies.

All procedures were done according to the technique described by Werner et al. [10] by one single surgeon [19] with the Delta components without changes of this procedure in the included patients. Although previous studies assumed possibilities to avoid infraglenoidal scapular notching the surgeon did not insert the metaglene more distally with respect to the findings of Nyfeller et al. [6] in any of the included cases.

Postoperatively, all patients began with immediate passive rehabilitation. In the first 6 postoperative weeks, patients used continuous passive motion (CPM) as well as free movements of the fingers and elbow joint in all directions where no weights were allowed. At the beginning of the 7^th ^week, patients moved their shoulder in all directions with light weights of a maximum of 12 pounds. After the 11^th ^week, patients were admitted unrestricted activity in all directions and to participate in sports with no high impact to the glenohumeral joint, such as running or cycling. This rehabilitation regimen was identical for all patients.

At a minimum follow-up of 2 years (mean 45 months, range 24 to 96 months), 60 patients (27 male and 33 female) at 67 years of mean age (range, 56 to 84 years) with a mean height of 160.89 cm (range: 148 to 175) and a mean weight of 72.15 kg (range: 42 to 105) were re-examined using the same clinical scores as preoperatively and the DASH score [18,20-22].

**Figure 1:**
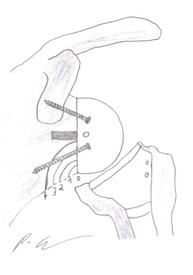
**The grade of infraglenoidal scapular notching after implantation of an inverted total shoulder prosthesis is classification by Nerot**. It is divided in "grade 0" for "no notch", "grade 1" for "small notch", "grade 2" for "notch with condensation", "grade 3" for "erosion up to the inferior screw", and "grade 4" for "erosion over the inferior screw with extension under the base plate".

Furthermore, all shoulders were analyzed in terms of anterior-posterior and axillary lateral radiographic views. These films were classified twice independently by two orthopaedic surgeons testing for indicators of infraglenoidal scapular notching according to the classification of Nerot [9] (Figure 1). Thereafter, infraglenoidal scapular notching was categorized as "grade 0" for "no notch", "grade 1" for "small notch", "grade 2" for "notch with condensation", "grade 3" for "erosion up to the inferior screw", and "grade 4" for "erosion over the inferior screw with extension under the base plate".

For the statistical evaluation of our findings, the radiological assessment according to Nerot et al. [9] was evaluated using a Cohen's kappa coefficient, which is a parameter of intra-observer agreement for continuous outcomes ranging from 1 (perfect agreement) to 0 (no agreement). The correlations stated in H_1 _and H_2 _were calculated using the Spearman coefficient between notching classified according to Nerot and the difference between postoperative and preoperative scores, except for the DASH score and the range of motion, which were recorded only postoperatively. We assumed a minimum effect size of 0.3, which is a low effect according to the classification by Cohen, to be of clinical relevance for the correlation of notching with clinical outcomes and subjective stability. In order to be able to show such an effect size with an alpha of 5% and a power of 80% a minimum sample size of 60 patients was required.

G*Power 3 was used for sample size calculations. The SPSS version 13.0 for Windows (SPSS Inc., Chicago, IL, USA) was used for statistical analysis which was performed with 2-tailed, independent t tests for normally distributed data and Mann- Whitney U tests for nonparametric data in the case of the preoperative and postoperative clinical scores. A *P*-value of less than 0.05 was considered to be significant.

In addition, we performed a systematic online literature search of related studies in PubMED using the terms "scapular notching", "inverted total shoulder arthroplasty" and "reverse shoulder prosthesis". We included and abstracted data from studies on the clinical effect of scapular notching at a minimum follow-up of two years. We excluded papers not providing detailed data on the correlation of infraglenoidal scapular notching with clinical outcome at a minimum follow-up of two years.

## Results

Results of the clinical and stability scores are shown in Table 1. At a mean follow-up time of 42 months the active range of motion significantly increased in terms of a mean active anterior elevation from 43.2 to 104.5 degrees and a mean active abduction from 44.3 to 98.7 degrees (p < 0.0033 and p < 0.046). Active external rotation showed no significant difference with the values from 14.5 to 14.1 degrees at p = 0.096. There was a significant improvement in the clinical and stability scores which is reported in Table 1.

**Table 1:** Comparison of preoperative clinical evaluation with postoperative outcome after implantation of an inverted total shoulder prosthesis Delta with significance levels

	Preoperative analysis	At last follow-up	*P*-value
Fup (24 - 90 months), n = 60			

Oxford instability score	21.8 (10 - 39)	36.9 (23 - 48)	*p *< 0.01

Rowe score for instability	50.2 (10 - 80)	82.1 (25 - 100)	*p *< 0.05

Constant pain score	4.5 (0 - 12)	11.2 (2 - 15)	*p *< 0.03

Constant shoulder score	32.9 (14 - 63)	63.4 (19 - 90)	*p *< 0.02

	Operated side	Contralateral side	

DASH score**	32.1 (5.8 - 69)	21.9 (3.3 - 64.7)	*p *> 0.65

We present mean values and ranges (in parentheses) of the obtained stability scores and clinical scores.

** The DASH score is the only score, which had not been obtained preoperatively. Thereafter, we provide data in comparison to the contralateral shoulder.

**Figure 2:**
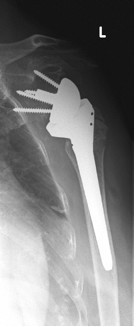
**Antero-posterior radiograph of a 56 year-old male patient ´ s left shoulder with an implanted inverted total shoulder prosthesis Delta at 52 months of follow-up**. Radiological analysis reveals "grade 2 = notch with condensation" of infraglenoidal scapular notching according to Nerot.

**Figure 3:**
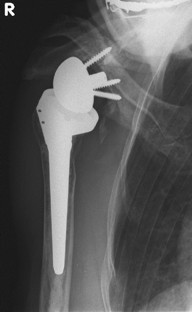
**Antero-posterior radiograph of a 64 year-old female patient ´ s right shoulder with an implanted inverted total shoulder prosthesis Delta at 39 months of follow-up**. Radiological analysis reveals "grade 4 = erosion over the inferior screw with extension under the base plate" of infraglenoidal scapular notching according to Nerot.

**Figure 4:**
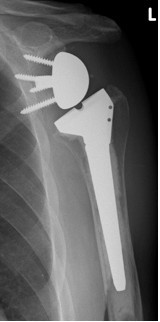
**Antero-posterior radiograph of a 74 year-old female patient ´ s left shoulder with an implanted inverted total shoulder prosthesis Delta at 64 months of follow-up**. Radiological analysis reveals with "grade 3 = erosion up to the inferior screw" of infraglenoidal scapular notching according to Nerot.

**Figure 5:**
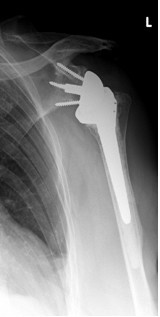
**Antero-posterior radiograph of a 71 year-old female patient ´ s left shoulder with an implanted inverted total shoulder prosthesis Delta at 31 months of follow-up**. Radiological analysis reveals "grade 4 = erosion over the inferior screw with extension under the base plate" of infraglenoidal scapular notching according to Nerot.

**Figure 6:**
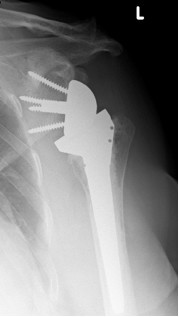
**Antero-posterior radiograph of a 75 year-old female patient ´ s left shoulder with an implanted inverted total shoulder prosthesis Delta at 32 months of follow-up**. Radiological analysis reveals "grade 4 = erosion over the inferior screw with extension under the base plate" of infraglenoidal scapular notching according to Nerot.

**Figure 7:**
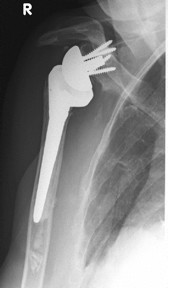
**Antero-posterior radiograph of a 69 year-old female patient ´ s right shoulder with an implanted inverted total shoulder prosthesis Delta at 44 months of follow-up**. Radiological analysis reveals with "grade 3 = erosion up to the inferior screw" of infraglenoidal scapular notching according to Nerot.

Radiological data in terms of infraglenoidal scapular notching which had been classified according to Nerot et al. [9] are presented in table 2 and figures 2, 3, 4, 5, 6, 7. Presented percentages correspond to the sum of measurements as two investigators evaluated X-rays twice resulting in 4 measurements for each patient and not to the single patients. The reliability of the radiological evaluation was evaluated by use of a Kappa coefficient of an "almost perfect" agreement with a value > 0.86.

**Table 2:** Grade of the infraglenoidal scapular notching after implantation of an inverted total shoulder prosthesis according to Nerot et al. ^16^

	Percentage	Corresponding notching according to Nerot et al. ^15^
MT - Fup, n = 48		

Grade 0	65%*	"no notch"

Grade 1	20%*	"small notch"

Grade 2	3%*	"notch with condensation"

Grade 3	6%*	"erosion up to the inferior screw"

Grade 4	6%*	"erosion over the inferior screw with extension under the base plate "

	Percentage	

LT - Fup, n = 12		

Grade 0	62%*	"no notch"

Grade 1	38%*	"small notch"

Grade 2	0%*	"notch with condensation"

Grade 3	0%*	"erosion up to the inferior screw"

Grade 4	0%*	"erosion over the inferior screw with extension under the base plate "

We divided these 60 patients in 48 patients with a mid-term (MT-Fup; 24-50 months) and 12 patients with a long-term follow-up (LT-Fup; 60-96 months). Infraglenoidal scapular notching was divided in "grade 0" for "no notch", "grade 1" for "small notch", "grade 2" for "notch with condensation", "grade 3" for "erosion up to the inferior screw", and "grade 4" for "erosion over the inferior screw with extension under the base plate"

* These percentages are calculated using the sum of four measurements in total (twice by two examiners).

With regard to H_1 _we found no significant correlation between objective infraglenoidal notching, classified by Nerot and subjective instability, measured by the Oxford instability score (p = 0.49) or the Rowe score for instability (p = 0.55). (Table 3)

**Table 3:** We correlated the grade of infraglenoidal scapular notching according to Nerot et al. after implantation of an inverted total shoulder prosthesis Delta with stability scores and clinical scores using a spearman correlation

**H**_**1**_	**Infraglenoidal notching according to Nerot et al.**^**16**^	*P*-value
**Fup (24-90 months), n = 60**		

Change of Oxford instability score	no significant correlation	0.49

Change of Rowe score for instability	no significant correlation	0.55

**H**_**2**_		

**MT-Fup (24-59 months), n = 48**		

Change of Constant pain score	no significant correlation	0.9018

Change of Constant shoulder score	no significant correlation	0.9546

Postoperative DASH score	no significant correlation	0.0819

Postoperative active anteversion	no significant correlation	0.4121

Postoperative active abduction	no significant correlation	0.4806

Postoperative external rotation	no significant correlation	0.4349

**LT-Fup (60-96 months), n = 12**		

Change of Constant pain score	**significant positive spearman correlation = 0.84**	0.0275

Change of Constant shoulder score	no significant correlation	0.8285

Postoperative DASH score	no significant correlation	0.3283

Postoperative active anteversion	**significant positive spearman correlation = 0.78**	0.0036

Postoperative active abduction	no significant correlation	0.7238

Postoperative external rotation	**significant positive spearman correlation = 0.91**	0.0008

These correlations were calculated using mean values of the mid-term (MT-Fup) and long-term follow-up (LT-Fup) groups. Note that we found a significant correlation of notching with clinical scores after dividing in a mid-term (24-60 months) and long-term follow-up (over 60 months).

For H_2 _we did not find any significant correlations at mid-term follow-up, ranging from 24 to 60 months, between infraglenoidal notching and objective clinical outcomes. In long-term follow-up (60 months and more) we found significant positive correlations between infraglenoidal notching and the Constant pain score (p = 0.3), and active anteversion (p < 0.01) and active external rotation (p < 0.01). These correlations are illustrated in table 3.

### Systematic review of the literature

With respect to the inclusion criteria of our systematic review of the literature we included 22 studies identified by the term "scapular notching", 134 studies identified by the term "reverse shoulder prosthesis", and further three studies identified by the term "inverted total shoulder arthroplasty". We excluded 155 studies with duplicates, irrelevant data of the correlation of infraglenoidal scapular notching and its correlation with clinical outcome at a minimum follow-up of 2 years. Thereafter, we present material of four studies from Lévigne et al. [5], Sirveaux et al. [8], Simovitch et al. [7], and Werner et al. [10], including data of 552 inverted total shoulder prosthesis according to our inclusion and exclusion criteria. Detailed information of these previous investigations and the authors' results is illustrated in Table 4.

**Table 4:** Review of the literature of Lévigne et al.,^5 ^Sirveaux et al.,^8 ^Simovitch et al.,^7 ^Werner et al.,^10 ^and the authors' results, which are divided in a mid-term follow-up (24 to 60 months) and a long-term follow-up (over 60 months) evaluating correlations with objective infraglenoidal notching and clinical results after implantation of an inverted total shoulder prosthesis

Study	Infraglenoidal scapular notching was correlated with	Findings
**Lévigne et al. **^**5 **^**(2008)**		

Follow-up (24-60 months)	Preoperative Constant shoulder score	No correlation

	Preoperative active range of motion	No correlation

	Postoperative Constant shoulder score	No correlation

	Postoperative active range of motion	No correlation

	Postoperative Constant pain score	No correlation

	Strength (part of the Constant shoulder score)	**Negative correlation**

**Sirveaux et al.**^**8 **^**(2004)**		

Follow-up (24-97 months)	Postoperative Constant shoulder score	**Negative correlation**

	Postoperative active range of motion	No correlation

**Simovitch et al**^**7 **^**(2007)**		

Follow-up (24-96 months)	Constant shoulder score	**Negative correlation**

	Subjective shoulder value	**Negative correlation**

	Active range of motion	**Negative correlation**

	Lower strength	**Positive correlation**

**Werner et al**^**10 **^**(2005)**		

Follow-up (over 24 months)	Constant shoulder score	No correlation

	Constant pain score	No correlation

	Active Range of motion	No correlation

**Own results (2010)**		

Follow-up (24-60 months)		

	Oxford instability score	No correlation

	Rowe score for instability	No correlation

	Constant pain score	No correlation

	Constant shoulder score	No correlation

	DASH score	No correlation

	Active range of motion	No correlation

Follow-up (over 60 months)		

	Oxford instability score	No correlation

	Rowe score for instability	No correlation

	Constant pain score	**Positive correlation**

	Constant shoulder score	No correlation

	DASH score	**Negative correlation**

	Active range of motion	**Positive correlation**

## Discussion

This study assessed the correlation between objective infraglenoidal notching and subjective stability and objective clinical outcome assessment at a minimum follow-up of two years after reverse total shoulder arthroplasty. Our study showed no significant correlation of the patients' subjective instability with objective infraglenoidal scapular notching. Furthermore, we did not find significant correlations of clinical parameters and scores with infraglenoidal scapular notching in the mid term, but in long-term follow-up over 60 months we observed a significant positive correlation of the Constant pain score and active range of motion, particularly anteversion and external rotation, with infraglenoidal notching, suggesting that may result in worse clinical outcome over time.

The results from the literature review widely corroborate our findings. While the results from Levigne et al. [5], Sirveaux et al. [8], Simovitch et al. [7], and our findings proved correlations of infraglenoidal scapular notching with clinical outcome, it is still unclear how to avoid this notching. We are in line with Levigne et al. [5] and Werner et al. [10] who stated that low positioning of the glenosphere is one of the most important factors to avoid scapular notching. Some authors propose to position the baseplate flush within the inferior glenoid rim so that the glenosphere extends 4 mm beyond the glenoid inferiorly [5,10]. This bares the risk of placement of the inferior screw below the scapular pillar or the superior screw beneath the base of the coracoid, which negatively affects the implant' s stability [5,10]. Another factor influencing scapular notching might be articular tension in the shoulder joint. Levigne et al. [5] observed less frequent notching in case of lateralized humeral cups than standard cups. They hypothesized, that thicker inserts results in higher articular and thereafter deltoid tension, which may limit arm adduction and lead to impingement and notching [5].

Various authors proposed other prosthetic designs to avoid a possible infraglenoidal scapular notching [5,6,23]. Frankle et al. [23] modified the mechanical concept of the Grammont prosthesis by lateralizing the center of its rotation. The benefit was less scapular notching but they observed a higher percentage of early baseplate fixation failures [23]. Our preference would be a humeral polyethylene cup with an asymmetric rim. These concepts have already been addressed by Nyffeller et al. [6] but there is a relatively high concern of a secondary prosthetic instability [5]. In contrast to that, Levigne et al. [5] propose to maintain the concept of the Grammont prosthesis and prevent the phenomenon of notching by different implantation devices.

Levigne et al. [5] stated that the craniocaudal position of the glenoid is essential for any possible progress of scapular notching. This is in line with Boileau et al. [1], Sirveaux et al. [16], Vanhove et al. [24], Werner et al. [10], and Nyffeller et al. [6] who demonstrated that a high placement of the glenoid implant favours scapular impingement and thereafter infraglenoidal notching in a Delta III prosthesis. According to Levigne et al. [5] a superior glenoid erosion is a predisposing factor for a too high positioning of the glenoid and therefore, they propose measuring the distance between the inferior glenoid bony rim and the lowest point of the glenosphere on a standardized anteroposterior radiograph.

The authors want to address the following potential limitations of their own results. The evaluation of radiological analysis was difficult because the notch might have been hidden by the glenosphere in case of no parallel beam to the baseplate in the frontal plane. Furthermore it might have been difficult to avoid superimposition of the ribs, especially in case of an anteverted baseplate. Next the authors only evaluated infraglenoidal scapular notching without looking at possible notching at the posterior glenoid. Furthermore, the Oxford instability score [18] and the Rowe score for instability [18] were designed to evaluate glenohumeral instability and not instability after implantation of prosthesis. Nevertheless, there are no scores to evaluate subjective instability in shoulder prosthesis and therefore these scores are most suitable.

In terms of the second study hypothesis patients with a long-term follow-up over 60 months were reported to have a significant positive correlation of the Constant pain score, and active anteversion and active external rotation with infraglenoidal notching. We have to address, that we do not believe that the relatively small number of patients with this follow-up (n = 12) can conclusively answer this question but our interpretation seems both biomechanically credible and biologically plausible.

However, the study strength has to be emphasised that we present a relatively large number of patients who all had been operated using the same technique and had the same postoperative and rehabilitation care. All patients had been clinically and radiologically analysed in terms of their notching and we evaluated our measurements by an inter- and intraobserver reproducibility.

## Conclusions

We conclude that patients' subjective impression on their shoulders' stability is not correlating with radiological signs of infraglenoidal scapular notching. Nevertheless we could demonstrate, that clinical parameters are affected by infraglenoidal scapular notching, at least in the long term.

## Competing interests

There exist no financial or non-financial competing interests in case of any author of this manuscript. No benefits or funds were received in support for the study.

## Authors' contributions

PS: preparation of the manuscript, data collection, study design; AL: revision of the manuscript, statistical advice; PV: revision of the manuscript, statistical analysis; AH: revision of the manuscript, statistical analysis; GW: data collection, study design, operating surgeon; JH: data collection, study design; MFP: revision of the manuscript; PEM: study design, revision of the manuscript. All authors read and approved the manuscript.

## Pre-publication history

The pre-publication history for this paper can be accessed here:

http://www.biomedcentral.com/1471-2474/12/101/prepub

## References

[B1] Boileau P Watkinson DJ Hatzidakis AM Balg F Grammont reverse prosthesis: design, rationale, and biomechanics J Shoulder Elbow Surg 2005 14 Suppl 1 147 61 10.1016/j.jse.2004.10.006 15726075

[B2] Boulahia A Edwards TB Walch G Baratta RV Early results of a reverse design prosthesis in the treatment of arthritis of the shoulder in elderly patients with a large rotator cuff tear Orthopedics 2002 25 129 33 11866145 10.3928/0147-7447-20020201-16

[B3] Delloye C Joris D Colette A Eudier A Dubuc JE Complications mécaniques de la prothése totale inversée de l'é paule Rev Chir Orthop 2002 88 410 4 12124542

[B4] De Wilde LF Plasschaert FS Audenaert EA Verdonk RC Functional recovery after a reverse prosthesis for reconstruction of the proximal humerus in tumor surgery Clin Orthop Relat Res 2005 430 156 62 15662318 10.1097/01.blo.0000146741.83183.18

[B5] Lévigne C Boileau P Favard L Walch G, Boileau P, Molé D, Favard L, Lévigne C, Sirveaux F Scapular notching in reverse shoulder arthroplasty Reverse shoulder arthroplasty 2006 Paris: Sauramps Mé dical 353 72

[B6] Nyffeler RW Werner CM Gerber C Biomechanical relevance of glenoid component positioning in the reverse Delta III total shoulder prosthesis J Shoulder Elbow Surg 2005 14 524 8 10.1016/j.jse.2004.09.010 16194746

[B7] Simovitch R Zumstein M Lohri E Helmy M Gerber C Predictors of scapular notching in patients managed with the Delta III reverse shoulder replacement J Bone Joint Surg Am 2007 89 588 600 10.2106/JBJS.F.00226 17332108

[B8] Sirveaux F Favard L Oudet D Huquet D Walch G Mole D Grammont inverted total shoulder arthroplasty in the treatment of glenohumeral osteoarthritis with massive rupture of the cuff. Results of a multicentre study of 80 shoulders J Bone Joint Surg Br 2004 86 388 95 10.1302/0301-620X.86B3.14024 15125127

[B9] Valenti PH Boutens D Nerot C Walch G, Boileau P, Molé D Delta 3 reversed prosthesis for osteoarthritis with massive rotator cuff tear: long term results 2000 shoulder prostheses ... two to ten year follow-up 2001 Montpellier, Paris, France: Sauramps Medical 253 9

[B10] Werner CM Steinmann PA Gilbart M Gerber C Treatment of painful pseudoparesis due to irreparable rotator cuff dysfunction with the Delta III reverse-ball-and-socket total shoulder prosthesis J Bone Joint Surg Am 2005 87 1476 86 10.2106/JBJS.D.02342 15995114

[B11] Farshad M Gerber C Reverse total shoulder arthroplasty-from the most to the least common complication Int Orthop 2010 34 8 1075 82 10.1007/s00264-010-1125-2 20865260 PMC2989053

[B12] John M Pap G angst F Flury MP Lieske S Schwyzer HK Simmen BR Short-term results after reversed shoulder arthroplasty Delta III in patients with rheumatoid arthritis and irreparable rotator cuff tear Int Orthop 2010 34 71 77 10.1007/s00264-009-0733-1 19221749 PMC2899269

[B13] Seebauer L Total reverse shoulder arthroplasty: European lessons and future trends Am J Orthop (Belle Mead NJ) 2007 36 Suppl 1 22 28 18264554

[B14] De Wilde L Mombert M Van Petegem P Verdonk R Revision of shoulder replacement with a reversed shoulder prosthesis (Delta III): report of five cases Acta Orthop Belg 2001 67 348 53 11725566

[B15] Nyffeler RW Werner CML Simmen BR Gerber C Analysis of a retrieved Delta III total shoulder prosthesis J Bone Joint Surg Br 2004 86-B 1187 91 10.1302/0301-620x.86b8.15228 15568535

[B16] Sirveaux F Favard L Oudet D Huquet D Walch G Mole D Grammont inverted total shoulder arthroplasty in the treatment of glenohumeral osteoarthritis with massive rupture of the cuff. Results of a multicentre study of 80 shoulders J Bone Joint Surg Br 2004 86 388 95 10.1302/0301-620X.86B3.14024 15125127

[B17] Sirveaux F Favard L Oudet D Huguet D Lautman S Walch G, Boileau P, Molé D Grammont inverted total shoulder arthroplasty in the treatment of glenohumeral osteoarthritis with massive and non repairable cuff rupture 2000 shoulder prostheses ... two to ten year follow-up 2001 Montpellier, Paris, France: Sauramps Medical 247 52

[B18] OrthopaedicScores http://www.orthopaedicscores.com/

[B19] Sadoghi p Vavken P Leithner A Hochreiter J Weber G Pietschmann MF Müller PE Impact of previous rotator cuff repair on the outcome of reverse shoulder arthroplasty J Shoulder Elbow Surg 2011 in press 10.1016/j.jse.2011.01.013 21454102

[B20] Dixon D Johnston M McQueen M Charles Court-Brown C The Disabilities of the Arm, Shoulder and Hand Questionnaire (DASH) can measure the impairment, activity limitations and participation restriction constructs from the International Classification of Functioning, Disability and Health (ICF) BMC Musculoskeletal Disorders 2008 9 114 10.1186/1471-2474-9-114 18715495 PMC2533660

[B21] Gummesson C Atroshi I Ekdahl C The disabilities of the arm, shoulder and hand (DASH) outcome questionnaire: longitudinal construct validity and measuring self-rated health change after surgery BMC Musculoskeletal Disorders 2003 4 11 10.1186/1471-2474-4-11 12809562 PMC165599

[B22] Gummesson C Ward MM Atroshi I The shortened disabilities of the arm, shoulder and hand questionnaire (*Quick*DASH): validity and reliability based on responses within the full-length DASH BMC Musculoskeletal Disorders 2006 7 44 10.1186/1471-2474-7-44 16709254 PMC1513569

[B23] Frankle M Siegal S Pupello D Saleem A Mighell M Vasey M The reverse shoulder prosthesis for glenohumeral arthritis associated with severe rotator cuff deficiency. A minimum two-year follow-up study of sixty patients J Bone Joint Surg Am 2005 87 1697 705 10.2106/JBJS.D.02813 16085607

[B24] Vanhove B Beugnies A Grammont's reverse shoulder prosthesis for rotator cuff arthropathy. A retrospective study of 32 cases Acta Orthop Belg 2004 70 219 25 15287400

